# The complete chloroplast genome sequences of Korean Native *Veronica* subgenus *Pseudolysimachion* species (Part II): *V. daurica* and *V. pyrethrina*

**DOI:** 10.1080/23802359.2026.2699475

**Published:** 2026-07-27

**Authors:** Sang Heon Kim, Ji Hun Yi, Jin-Woo Kim, Wonwoo Cho, Ji Young Jung

**Affiliations:** Forest Biological Resources Utilization Center, Korea National Arboretum, Yangpyeong, Republic of Korea

**Keywords:** Chloroplast genome, *Veronica daurica*, *Veronica pyrethrina*, Veroniceae, Plantaginaceae

## Abstract

We characterized the complete chloroplast genomes of *Veronica daurica* and *V. pyrethrina* (Plantaginaceae) using Illumina sequencing. The circular genomes were 152,223 bp and 152,240 bp in length, respectively, exhibiting a quadripartite structure with a large single-copy region (83,165–83,182 bp), a small single-copy region (17,700 bp), and a pair of inverted repeat regions (25,679 bp). Both genomes encoded 133 genes with a GC content of 38.0%. Maximum likelihood phylogenetic analysis revealed that *V. daurica* and *V. pyrethrina* form a monophyletic clade, showing a robust sister relationship with *V. longifolia* and *V. pusanensis*. These genomic resources, along with distinct SSR and RSCU profiles, resolve subgeneric morphological ambiguities and support future evolutionary studies.

## Introduction

The taxonomic classification of the tribe Veroniceae (Plantaginaceae) has been debated, particularly regarding the generic delimitation of *Pseudolysimachion* Opiz. Traditionally treated as an independent genus, it is now broadly reclassified as *Veronica* subgenus *Pseudolysimachion* (Koch) Alefeld based on phylogenetic and morphological evidence (Albach et al. 2004; Albach 2008). In the Korean Peninsula, this subgenus is represented by 17 native taxa with significant morphological diversity. Following our previous characterization of *V. longifolia* and *V. pusanensis* (Part I: Kim et al. 2026), we continue this series by investigating two additional Korean native species: *V. daurica* and *V. pyrethrina*.

*Veronica daurica* Steven (1817) (Dahurian spike speedwell; Gu-wa-kko-ri-pul) and *Veronica pyrethrina* Nakai (1943) (Large spike speedwell; Keun-gu-wa-kko-ri-pul) are perennial herbs representing another significant pair within the Korean *Pseudolysimachion*. Their distribution pattern parallels the relationship between *V. longifolia* and *V. pusanensis* described in our previous report (Part I). While *V. daurica* is widely distributed across East Asia, *V. pyrethrina* is a rare endemic species restricted to rocky habitats in central Korea. This contrast between a widespread taxon and a narrow endemic provides a unique opportunity to explore genomic divergence within the subgenus. Despite differing ranges, overlapping traits like spiked inflorescences often complicate precise identification.

The chloroplast (cp) genome serves as a high-resolution tool for resolving taxonomic ambiguities (Gitzendanner et al. 2018). While plastomes of several *Pseudolysimachion* species, such as *V. nakaiana* and *V. ovata*, have been reported (Choi et al. 2016; Maurya et al. 2020), complete genomic resources for *V. daurica* and *V. pyrethrina* remain unavailable. Characterizing these species is crucial for clarifying their phylogenetic positions and establishing a genomic foundation for conservation and ornamental resource development (Kim and Cho 2024).

In this second part, we present their complete chloroplast genomes to characterize their plastome structures, compare genomic features with previously reported *Veronica* species, and determine their phylogenetic relationships within Veroniceae.

## Materials and methods

### Plant material, DNA extraction, and sequencing

**Figure 1.**
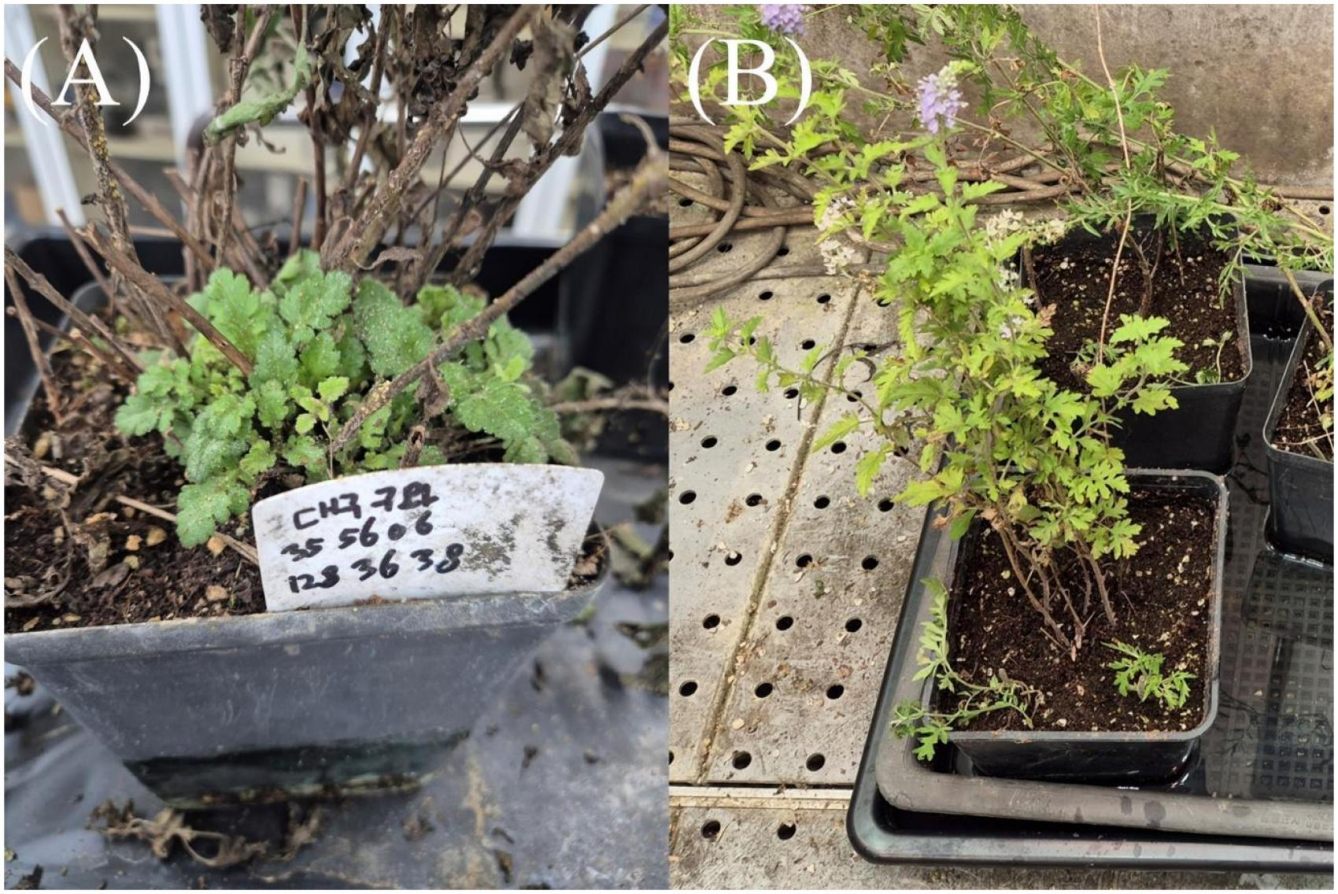
Photographs of Dahurian spike speedwell (*Veronica daurica*) (A) and large spike speedwell (*Veronica pyrethrina*) (B) used for the chloroplast genome assembly in this study. The sample numbers are FBRUC2025SHK00E and FBRUC2025SHK00G, respectively (photos taken by Sang Heon Kim at a greenhouse and a phyto-garden system at the Forest Biological Resources Utilization Center of the Korea National Arboretum).

*V. daurica* (Voucher KHB1663390) was gathered from Buk-gu, Daegu, Korea (35°56′06.2ʺN 128°36’37.9ʺE), and *V. pyrethrina* (KHB1663392) was obtained from Uiseong-gun, Gyeongsangbuk-do, Korea (36°13′46.6ʺN 128°45′17.7ʺE). Species identification was performed using the taxonomic keys from the Flora of Korea (Park 2018). The gathered plants were grown at the Forest Biological Resources Utilization Center of the Korea National Arboretum (Figure 1). Specimens were saved at the Herbarium of the Korea National Arboretum (Dr. Dong Chan Son, sdclym@korea.kr) with voucher numbers KHB1663390 and KHB1663392, respectively. Total genomic DNA was extracted from fresh leaf tissue using a DNeasy Plant Mini Kit (Qiagen, Valencia, CA, USA) according to the manufacturer’s instructions. Sequencing was conducted on the Illumina NovaSeq X platform (Illumina, San Diego, CA, USA) at Phyzen (Korea).

The genome assembly and annotation processes followed the methodologies described in our previous study (Part I: Kim et al. 2026). Briefly, the paired-end reads (mean length 151 bp) were assembled using NOVOPlasty v4.3.5 (Dierckxsens et al. 2017) with the *rbcL* gene from *V. nakaiana* (NC_031153) as a seed. The circularity and quadripartite structure of the assembled plastomes were validated through read-mapping using BWA-MEM. The final chloroplast genome sequences were annotated with GeSeq (Tillich et al. 2017) and visualized with OGDRAW (Greiner et al. 2019). To ensure accurate annotation, the detailed features of *cis-* and *trans-*splicing genes were analyzed and visualized using the CPGView pipeline (Liu et al. 2023), as presented in Supplementary Figures S2 and S3. This pipeline was deployed primarily to verify baseline structural integrity rather than to declare macro-evolutionary shifts.

**Figure 2.**
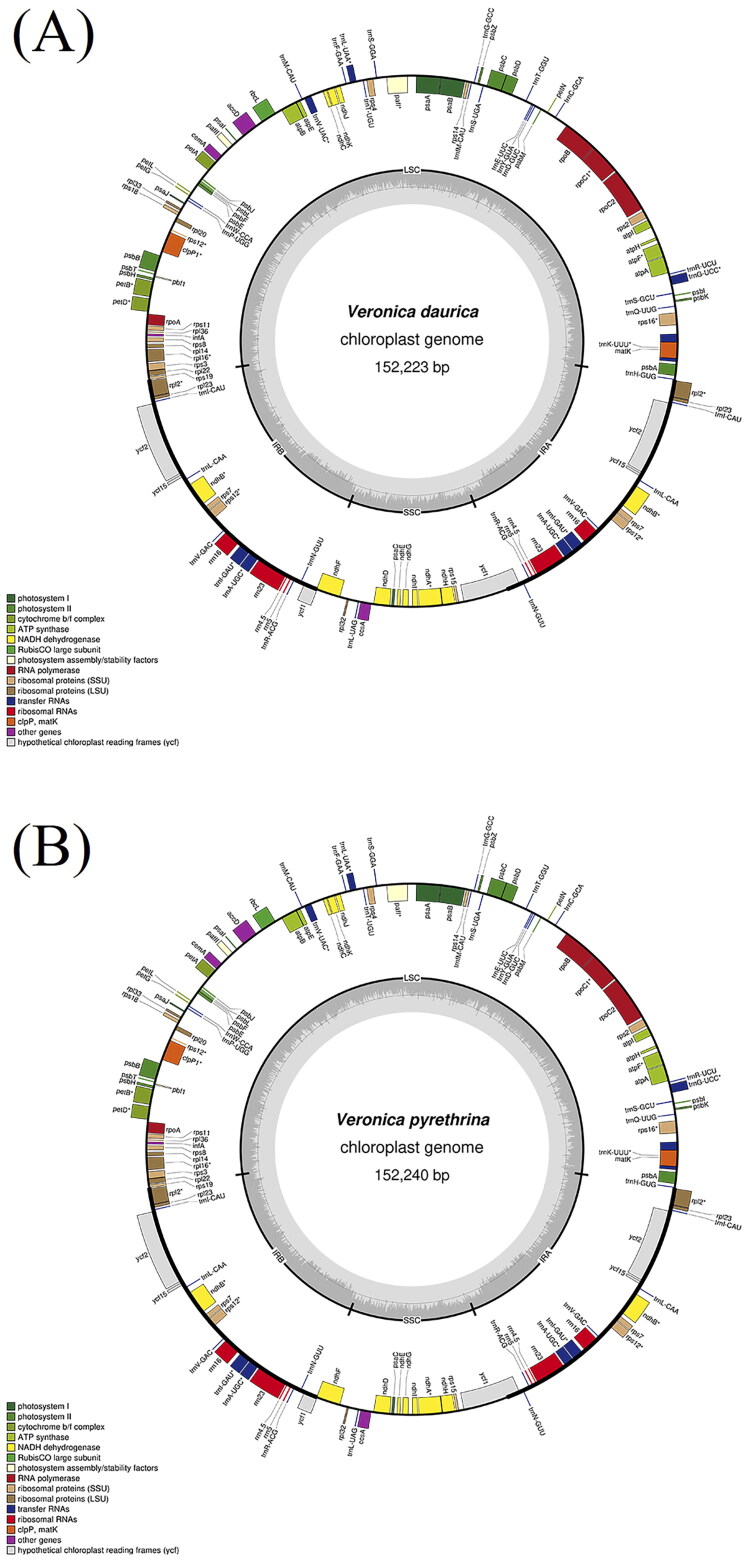
Complete chloroplast genome map of Dahurian spike speedwell (*Veronica daurica*) (A) and large spike speedwell (*Veronica pyrethrina*) (B). Genes shown on the outside of the circle are transcribed clockwise, while those on the inside are transcribed counterclockwise. The inner circle displays GC content (dark gray) and AT content (light gray). The large single-copy (LSC) and small single-copy (SSC) regions are separated by inverted repeat A (IRA) and inverted repeat B (IRB) regions.

**Figure 3.**
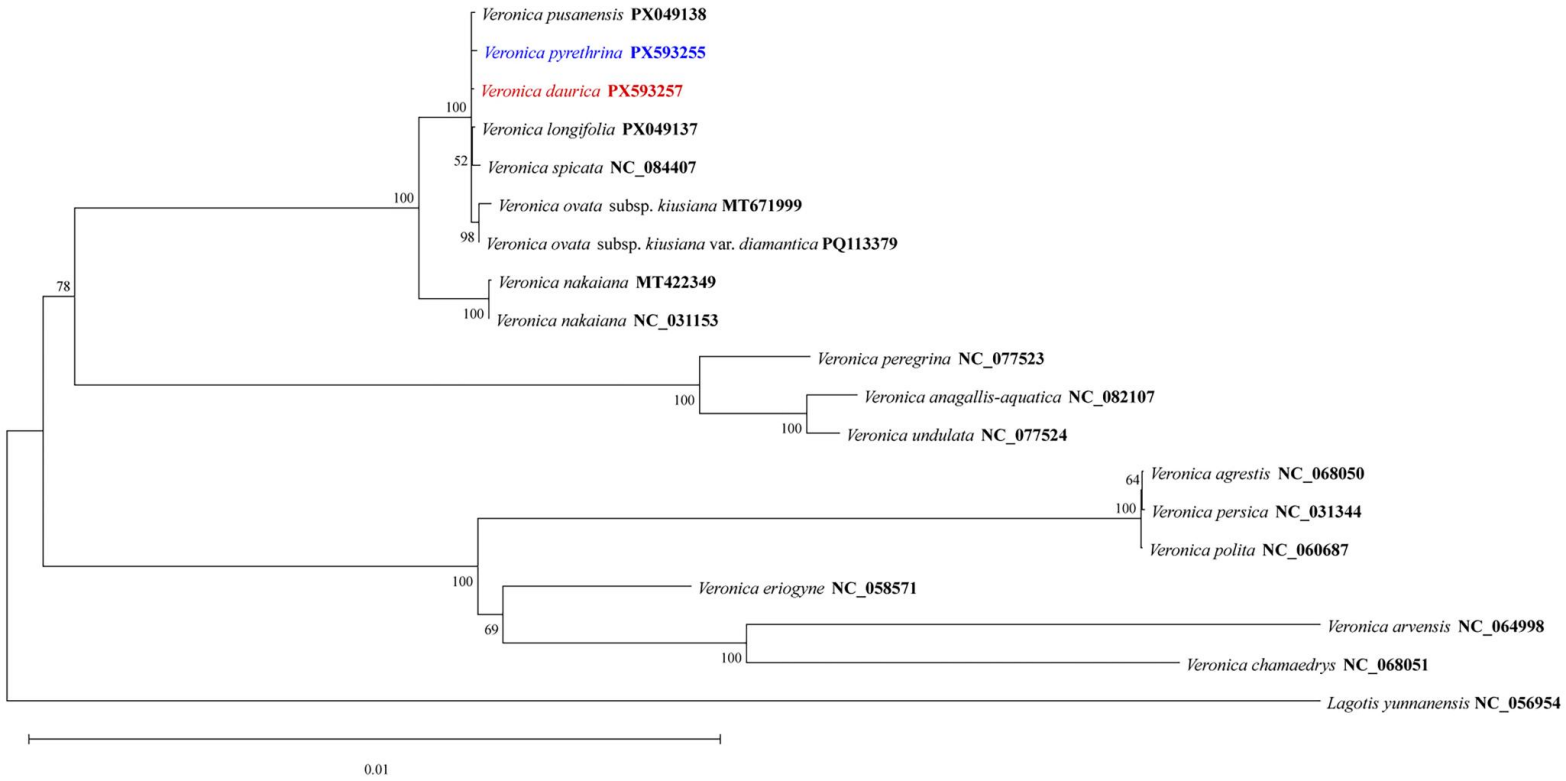
Maximum likelihood (ML) phylogenetic tree of the tribe Veroniceae based on complete chloroplast genome sequences. Bootstrap support values (>50%) are shown at each node. *Veronica daurica* PX593257 and *Veronica pyrethrina* PX593255, newly sequenced in this study, are highlighted in red and blue, respectively. The following sequences were used for comparative analysis: *Veronica longifolia* PX049137 (Part I: Kim et al. 2026), *Veronica pusanensis* PX049138 (Part I: Kim et al. 2026), *Veronica spicata* NC_084407, *Veronica ovata* subsp. *kiusiana* MT671999 (Maurya et al. 2020), *Veronica ovata* subsp. *kiusiana* var. *diamantiaca* PQ113379 (Ha et al. 2025), *Veronica nakaiana* MT422349 (Lee et al. 2021) and NC_031153 (Choi et al. 2016), *Veronica peregrina* NC_077523, *Veronica anagallis-aquatica* NC_082107 (Hai et al. 2024), *Veronica undulata* NC_077524, *veronica agrestis* NC_068050 (Zhao et al. 2023), *Veronica persica* NC_031344 (Choi et al. 2016), *Veronica polita* NC_060687 (Jia et al. 2022), *Veronica eriogyne* NC_058571 (Danzeng et al. 2021), *Veronica arvensis* NC_064998 (Liu et al. 2022), *Veronica chamaedrys* NC_068051 (Zhao et al. 2023). *Lagotis yunnanensis* NC_056954 (Cheng et al. 2020) was used as an outgroup. The scale bar indicates the number of nucleotide substitutions per site.

### Phylogenetic analysis

To determine the phylogenetic positions of *V. daurica* and *V. pyrethrina*, we conducted a comparative analysis using 17 additional complete chloroplast genome sequences from NCBI GenBank, including *V. longifolia* and *V. pusanensis* reported in Part I. *Lagotis yunnanensis* (NC_050965) was used as the outgroup based on tribal-level relationships within Plantaginaceae to ensure a stable root. Following the protocol established by Kim et al. (2026), protein-coding sequences were compiled and concatenated into a single dataset via PhyloSuite v1.2.2 (Zhang et al. 2020) and aligned with MAFFT v7.313 (Katoh and Standley 2013). A maximum likelihood (ML) tree was reconstructed using MEGA 12 (Kumar et al. 2024) with the GTR+I model and 1,000 bootstrap replicates. The maximum likelihood analysis *via* MEGA 12 provides sufficient topological reliability for the scale of our current taxon sampling.

### SSR and codon usage bias analysis

To address the structural characteristics and evolutionary constraints within the subgenus *Pseudolysimachion*, simple sequence repeats (SSRs) and codon usage patterns were comprehensively analyzed for four taxa: the two newly sequenced species (*V. daurica* and *V. pyrethrina*) and two previously reported relatives from Part I (*V. longifolia* and *V. pusanensis*). Characterization and genomic distribution of SSRs were performed using the CPStools pipeline (Huang et al. 2024). The SSR loci were screened and explicitly categorized based on their structural locations into coding genes, introns, and intergenic spacer (IGS) regions to evaluate dynamic sequence variations across the plastoms.

Furthermore, the relative synonymous codon usage (RSCU) was calculated using the same CPStools suite to determine linege-specific codon preference profiles. The synonymous codon preferences and frequency shifts among the four species were quantified and compared. To eliminate potential misinterpretation from conventional charts, the comparative codon usage bias profiles were visualized using the dedicated web-based interactive application RSCU-Plot (https://pcg-lab.shinyapps.io/RSCU-Plot/), which effectively illustrates the partitioned distribution of synonymous codons for each amino acid (Akrami et al. 2025; Diani Gohar and Soorni 2025; Hejazi et al. 2025; Soorni and Golchini 2025).

## Results

### General characteristics of the chloroplast genomes

The complete chloroplast (cp) genome sequences of *Veronica daurica* (GenBank accession no. PX593257) and *Veronica pyrethrina* (GenBank accession no. PX593255) were successfully determined, yielding circular molecules of 152,223 bp and 152,240 bp, respectively (Figure 2, Table 1). Both plastomes maintained the conserved quadripartite architecture, a hallmark of the subgenus *Pseudolysimachion*, comprising a Large Single-Copy (LSC) region, a Small Single-Copy (SSC) region, and two Inverted Repeats (IRs). Specifically, the LSC spanned 83,165–83,182 bp, while the SSC and IRs were identical in length for both species at 17,700 bp and 25,679 bp, respectively.

**Table 1.** Comparison of general characteristics of *Veronica daurica* and *Veronica pyrethrina.*

	*Veronica daurica*	*Veronica pyrethrina*
Sequencing information (Pair-end reads)		
Raw data read number	45,935,514	45,332,654
Mapped read number	9,917,484	18,266,388
Chloroplast coverage (×)	9764.17	180537.9
Chloroplast genome size (bp)		
Total genome	152,223	152,240
Large single-copy	83,165	83,182
Small single-copy	17,700	17,700
Inverted repeat	25,679	25,679
GC content (%)		
Total genome	38.0	38.0
LSC	36.1	36.1
SSC	31.9	31.9
IR	43.2	43.2
Number of genes		
Total	133	133
Protein-coding	88	88
tRNA	37	37
rRNA	8	8

The assembly quality was validated by high sequencing depths, reaching 9764.17× for *V. daurica* and a significantly higher 180537.9× for *V. pyrethrina* (Table 1, Figure S1). This variation in depth is likely attributable to the fluctuating concentration of plastid DNA within the total genomic extract of each sample. The total GC content remained stable at 38.0% across both taxa. Within the subregions, GC distribution followed a predictable pattern: 43.2% in the IRs, 36.1% in the LSC, and 31.9% in the SSC. Consistent with other *Veronica* species, each genome encoded 133 genes, including 88 protein-coding genes (PCGs), 37 transfer RNAs (tRNAs), and 8 ribosomal RNAs (rRNAs). Genomic architecture, including the presence of 13 *cis-*splicing genes (Figure S2) and one *trans-*splicing gene (*rps12*; Figure S3), was strictly conserved.

### Phylogenetic analysis

To investigate the evolutionary placement of the studied taxa within the tribe Veroniceae, a Maximum Likelihood (ML) phylogenetic analysis was performed (Figure 3). Within this framework, the 18 included taxa of the tribe Veroniceae (with *Lagotis yunnanensis* as the outgroup) were resolved into distinct lineages. In agreement with previous molecular evidence (Albach 2008, Albach et al. 2004), our findings demonstrate that the genus *Veronica* is polyphyletic. Specifically, the subgenus *Pseudolysimachion* emerged as a clearly defined monophyletic group embedded within the broader *Veronica* radiation, rather than being a sister lineage to the genus as a whole.

Within the *Pseudolysimachion* cluster, the newly sequenced *V. daurica* and *V. pyrethrina* were nested in a highly supported subclade (BS = 100) alongside *V. longifolia* and *V. pusanensis*. Notably, the Korean endemic *V. pyrethrina* exhibited a close sister relationship with *V. pusanensis* (BS = 100), while *V. daurica* was grouped with *V. longifolia* (BS = 78). This arrangement provides further genomic evidence for the close affinity among these East Asian *Pseudolysimachion* species, clearly distinguishing them from the *V. ovata* complex and *V. spicata* lineages.

### Simple sequence repeats (SSRs) and codon usage bias characterization

A comparative screen using CPStools identified 42, 40, 42, and 46 SSRs in the plastoms of *V. daurica*, *V. pyrethrina*, *V. longifolia*, and *V. pusanensis*, respectively (Table 2). Coding-embedded SSRs were strictly conserved at three loci across all taxa (within *rpoC2*, *atpB*, and *rpoA*). In non-coding regions, intron-embedded SSRs displayed a dimorphic pattern: 7 loci in *V. daurica* and *V. pusanensis*, and 6 loci in *V. pyrethrina* and *V. longifolia*. Variation in total SSR counts was primarily driven by the dynamic intergenic spacer (IGS) regions, ranging from 31 (*V. pyrethrina*) to 36 loci (*V. pusanensis*).

**Table 2.** Comparative analysis of simple sequence repeats (SSRs) distribution among four *Veronica* subgenus *Pseudolysimachion* chloroplast genomes.

Region (Loc type)	*V. daurica*	*V. pyrethrina*	*V. longifolia*	*V. pusanensis*
Gene (coding)	3	3	3	3
Intron	7	6	6	7
IGS (Intergenic)	32	31	33	36
Total SSRs	42	40	42	46

The relative synonymous codon usage (RSCU) analysis revealed highly congruent codon preferences among the four species, showing a typical bias toward A/T-ending codons (Supplementary Figure S4). The Part I species (*V. longifolia* and *V. pusanensis*) shared an identical RSCU matrix, while the newly sequenced Part II species (*V. daurica* and *V. pyrethrina*) clustered as a synchronized group. Minor but distinct micro-evolutionary shifts in RSCU values were detected between the Part I and Part II lineages, such as the GGT (Gly) codon shifting from 1.29 to 1.28, and the ACA (Thr) codon increasing from 1.22 to 1.24.

## Discussion and conclusion

This study offers significant genomic perspectives on the evolutionary framework of the tribe Veroniceae, with a specific focus on the taxonomic resolution of *Veronica* subgenus *Pseudolysimachion*. The phylogenetic analysis based on whole chloroplast genomes robustly confirmed the monophyletic status of the Plantaginaceae, consistent with previous molecular assessments (Albach et al. 2004; Maurya et al. 2020).

The freshly assembled plastomes of *V. daurica* and *V. pyrethrina* provided crucial evidence to reinforce the monophyly of the subgenus *Pseudolysimachion*. Our phylogenetic data demonstrate that these taxa are deeply nested within the genus *Veronica*, explicitly refuting the earlier hypothesis that *Pseudolysimachion* acts as a sister lineage to the entire genus (Albach 2008). Notably, the close phylogenetic affinity between *V. daurica* and *V. longifolia*, as well as the sister relationship between the Korean endemic *V. pyrethrina* and *V. pusanensis*, suggests a shared evolutionary trajectory among these East Asian species. The robust grouping of these four taxa indicates that their divergence might be linked to the complex biogeographical history of the Korean Peninsula and neighboring regions. Our CPStools dataset refines this evolutionary context and addresses concerns regarding sequential novelty over Part I. Despite sharing 42 total SSRs, *V. daurica* possesses 7 intron-embedded and 32 IGS SSRs compared to the 6 intron and 33 IGS SSRs in *V. longifolia*, providing a clear structural basis for their divergence (Table 2). Furthermore, systematic codon preference shifts explicitly separate the two study groups (Supplementary Figure S4); the newly sequenced Part II lineages exhibit a slight reduction in GGT (Gly) and AGG (Arg) preferences alongside an elevation in CGA (Arg) and ACA (Thr) values. These coordinated substitutions, coupled with the unique SSR contraction (40 loci) in the rare endemic *V. pyrethrina*, demonstrate that these plastoms have accumulated distinct, lineage-specific evolutionary constraints post-speciation.

This sequential framework progresses from Part I by shifting focus to the subgeneric clade characterized by deeply incised or pinnately cleft leaf morphology. Characterizing *V. pyrethrina*—a rare endemic species with a 180,537.9× sequencing depth—provides irreplaceable conservation genetic resources for East Asian flora. Furthermore, the identical IR length (25,679 bp) highlights high genomic stability post-speciation. These newly identified polymorphic sites offer robust SNPs and molecular IDs to resolve taxonomic ambiguities among morphologically cryptic *Veronica* species. These genomic resources now clarify the long-debated taxonomic positions of these species and provide a foundational dataset for understanding speciation within the subgenus.

While our plastid-based analysis establishes a reliable phylogenetic framework, it is important to recognize the limitations of single-locus, maternally inherited data. Potential evolutionary complexities such as hybridization or incomplete lineage sorting can lead to discrepancies between plastid and nuclear evolutionary histories, a phenomenon increasingly recognized in recent phylogenomic studies (Stull et al., 2020). Therefore, to better account for these complexities, subsequent research should integrate multi-locus nuclear markers and expanded taxon sampling. Such an integrative approach will provide a higher-resolution understanding of the reticulate evolution and biogeographical diversification of *Veronica* species in East Asia.

## Supplementary Material

Supplementary_Figures_Clean.docx

## Data Availability

The data obtained from this study are openly available in GenBank of NCBI at https://www.ncbi.nlm.nih.gov/ under the accession number PX593257 and PX593255. The associated BioProject, SRA, and BioSample numbers are PRJNA1295692, SRR35940834 and SRR35940832, and SAMN52949799 and SAMN52949801, respectively.
