# Supplementary material for: The complete chloroplast genome sequences of Korean Native Veronica subgenus Pseudolysimachion species (Part II): V. daurica and V. pyrethrina
Source: Mitochondrial DNA B Resour. 2026 Jul 27;11(9):973–8. doi: 10.1080/23802359.2026.2699475 (PMC13410545; doi:10.1080/23802359.2026.2699475)
Supplement: Supplementary_Figures_Clean.docx [file TMDN_A_2699475_SM7838.docx]

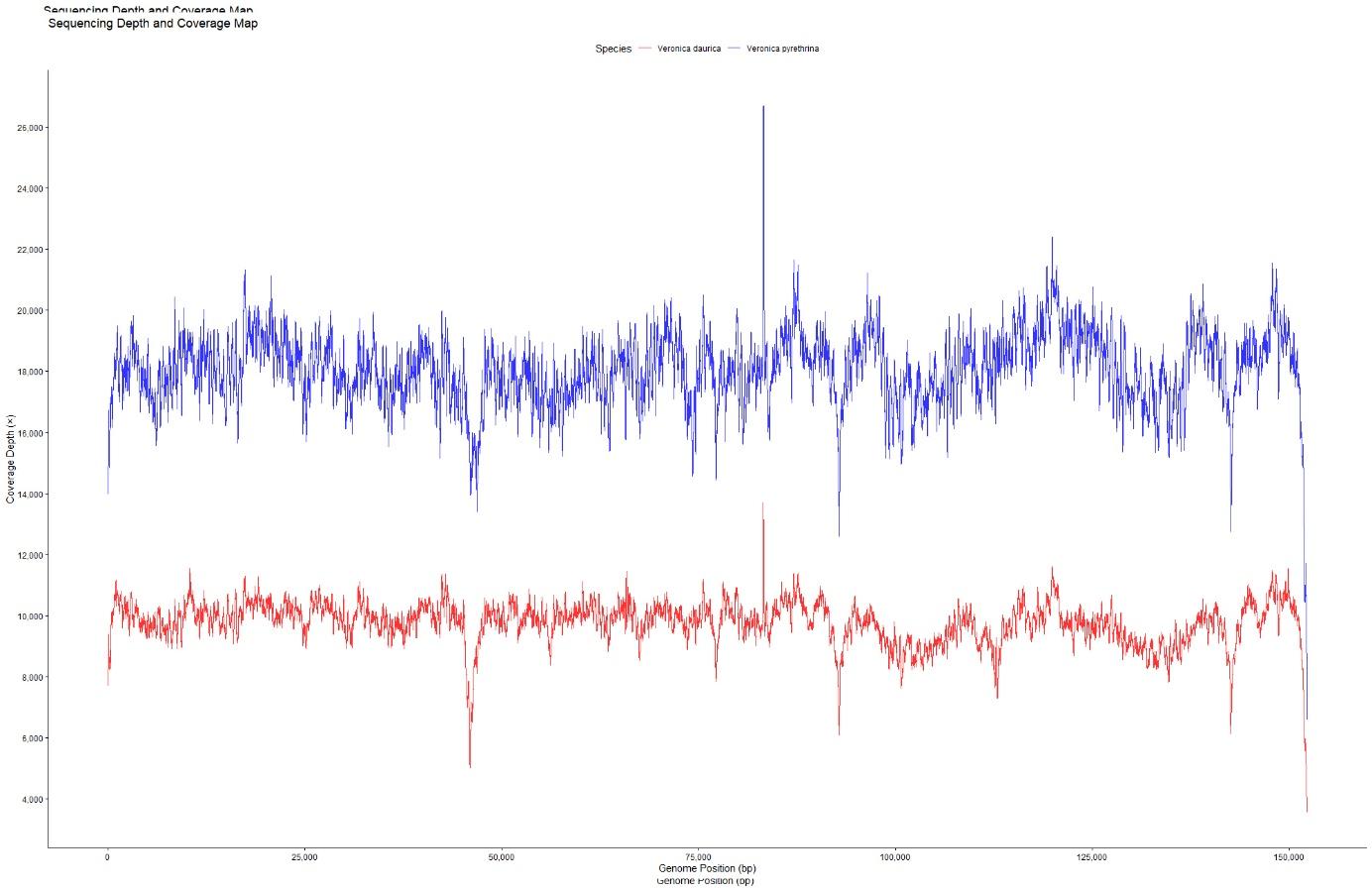


**Figure S1.** Sequencing depth and coverage map of *Veronica daurica* and *V. pyrethrina*. The red line indicates the coverage depth of *V. daurica* and the blue line indicates the coverage depth of *V. pyrethrina*. The average coverages were 9764.17× and 180537.9×, respectively. The X-axis represents the nucleotide position of the chloroplast genome, and the and Y-axis represents the read mapping depth.


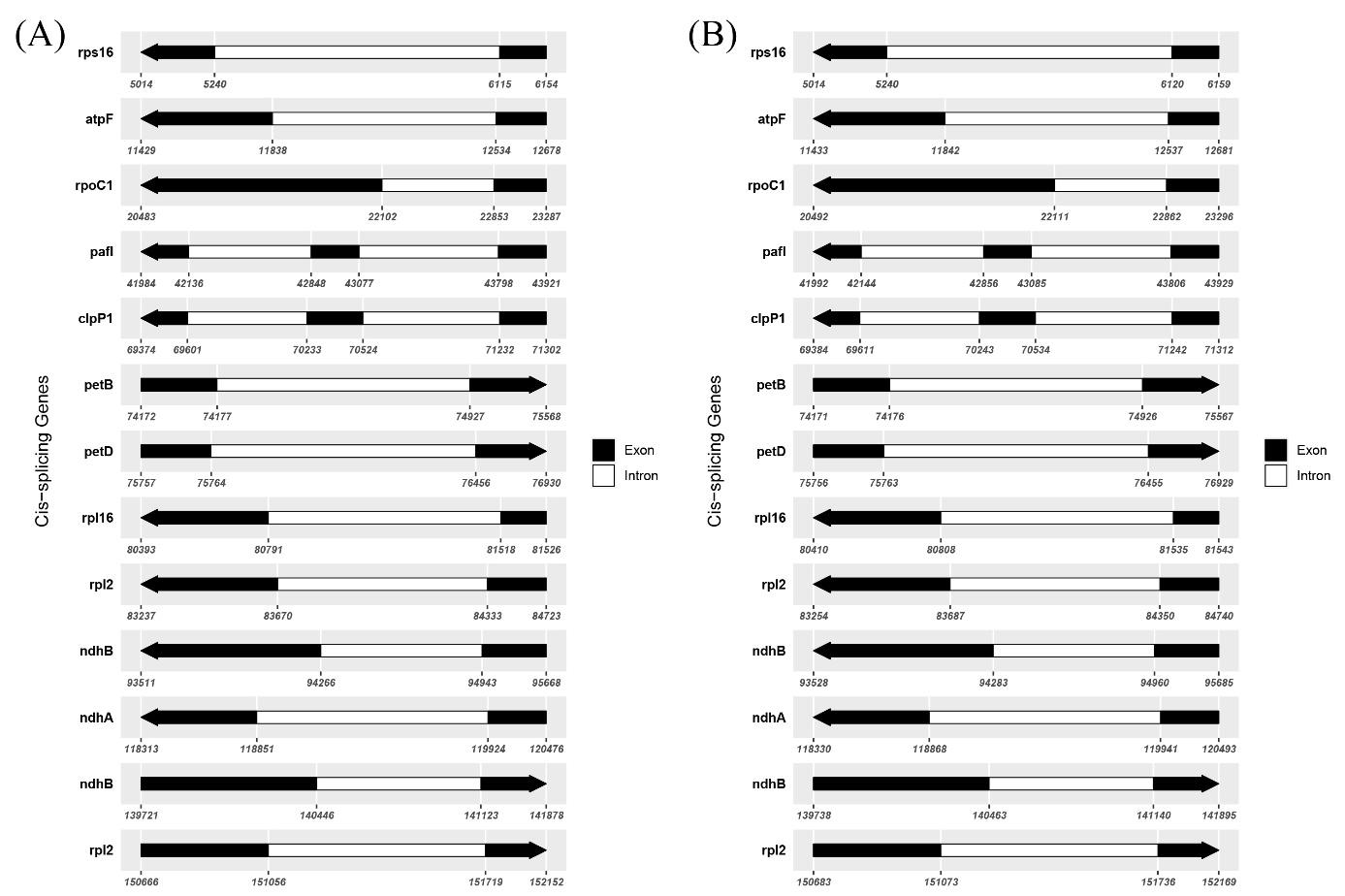


**Figure S2.** Schematic maps of cis-splicing genes in the chloroplast genome of Dahurian spike speedwell (*Veronica daurica*) (A) and Large spike speedwell (*V. pyrethrina*) (B). The large arrows represent the cis-spliced genes, with the arrow direction indicating the gene strand. Exons are shown as black rectangles and introns as white rectangles within the genes. This figure highlights the gene structure, including exon-intron boundaries, for a subset of genes.


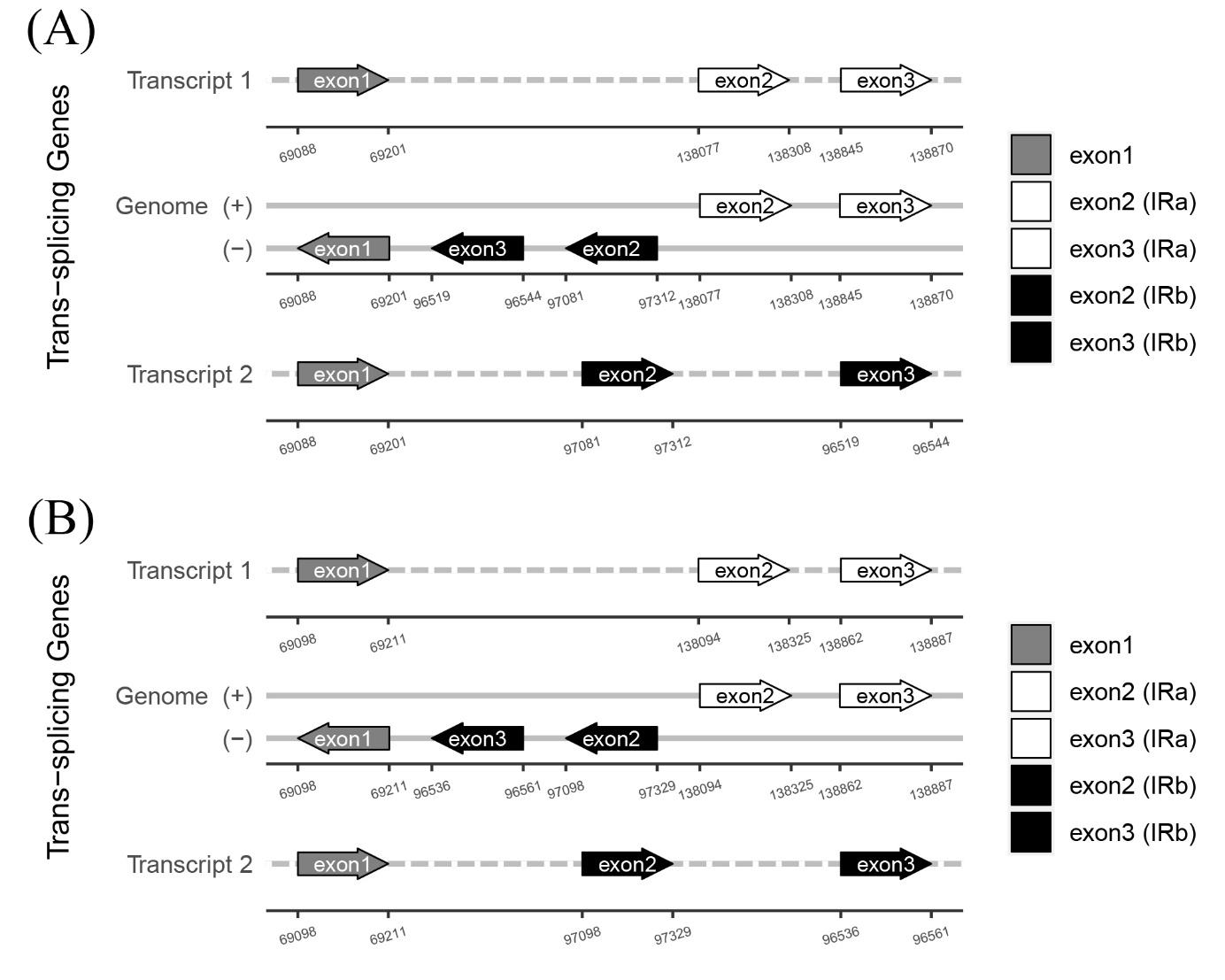


**Figure S3.** Schematic maps of the trans-splicing gene the chloroplast genomes of Dahurian spike speedwell (*Veronica daurica*) (A) and Large spike speedwell (*V. pyrethrina*) (B). The gene *rps12* is presented as a representative trans-splicing gene, where its exons are transcribed from two separate locations in the genome and later joined together. The colors in the legend indicate the different exons and their locations in the inverted repeat (IR) regions. The arrows and dashed lines illustrate the transcript joining process.


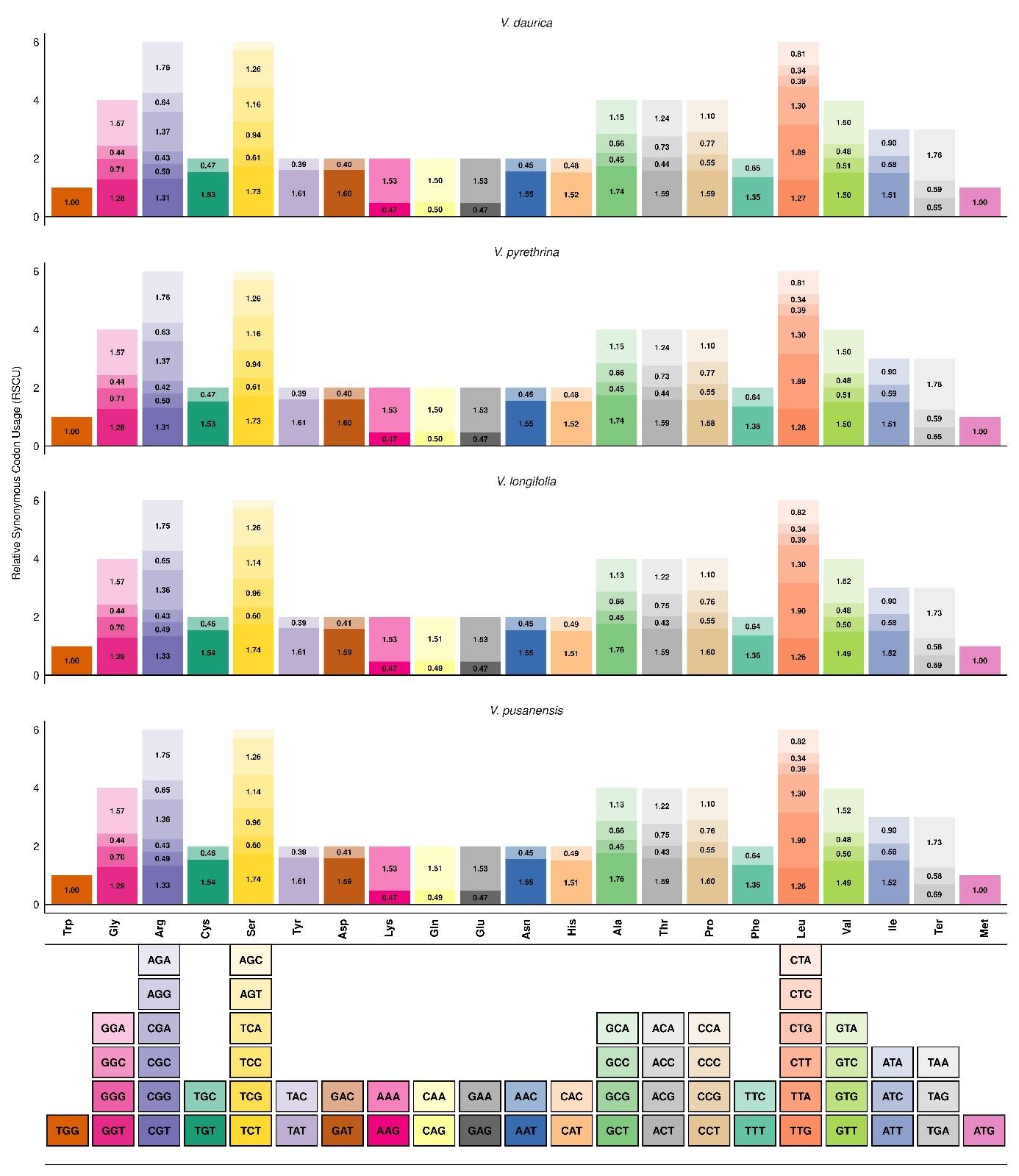


**Figure S4.** Comparative analysis of Relative Synonymous Codon Usage (RSCU) in four *Veronica* subgenus *Pseudolysimachion* chloroplast genomes. Codons are grouped by their corresponding amino acids along the x-axis, with the colored segments representing individual synonymous codons to illustrate relative usage bias.
